# The application of lasers in vital pulp therapy: clinical and radiographic outcomes

**DOI:** 10.1186/s12903-024-04026-x

**Published:** 2024-03-14

**Authors:** Farzaneh Afkhami, Golriz Rostami, Chun Xu, Ove A. Peters

**Affiliations:** 1 School of Dentistry, Tehran University of Medical Sciences, Tehran, Iran; 2 School of Dentistry, The University of Queensland, Brisbane, Australia; 3 Private Practice, Toronto, Canada

**Keywords:** Dental pulp, Dental pulp capping, Lasers, Treatment outcome, Radiography, Radiographic image interpretation

## Abstract

The main purpose of vital pulp therapy (VPT) is to preserve the integrity and function of the pulp. A wide variety of materials and techniques have been proposed to improve treatment outcomes, and among them, the utilization of lasers has gained significant attention. The application of lasers in different stages of VPT has witnessed remarkable growth in recent years, surpassing previous approaches.

This study aimed to review the applications of lasers in different steps of VPT and evaluate associated clinical and radiographic outcomes. An electronic search using Scopus, MEDLINE, Web of Science and Google Scholar databases from 2000 to 2023 was carried out by two independent researchers. The focus was on human studies that examined the clinical and/or radiographic effects of different laser types in VPT. A total of 4243 studies were included in this narrative review article. Based on the compiled data, it can be concluded that although current literature suggests laser may be proposed as an adjunct modality for some procedural steps in VPT, more research with standardized methodologies and criteria is needed to obtain more reliable and conclusive results.

## Background

Vital pulp therapy (VPT) refers to preservation of the vitality and function of the pulp in cases with compromised pulp tissue due to caries, restorative procedures, or trauma [1]. Preservation of pulp vitality decreases the hard tissue removal, maintains dentin deposition, pulpal immunological response as well as proprioceptive functions [2]. Several materials and techniques have been proposed for VPT over the years. Lasers application in VPT is among the relatively novel modalities. Many histological, clinical, and radiographic reports are available regarding the application of lasers for dental pulp-related treatment procedures.

VPT should be initiated by preparing the dentin at the site of pulp exposure. All caries should be removed with manual instruments or high- or low-speed handpieces [3]. Laser irradiation has been proposed for this step. The advantages of tooth preparation with laser include reduced need for anesthesia [4, 5], lower perioperative pain, and lower level of stress especially in pediatric patients [5–7]. Of different laser types, currently, only erbium lasers have the potential for use in cavity preparation. Since such lasers are not in contact with the target tissue, they cause minimal mechanical trauma [3] and lower temperature rise in the underlying pulp tissue compared with rotary handpieces [8]. Lasers can also be effectively used for the removal of smear layer caused by cavity preparation with a handpiece [9–11].

The creation of a sterile zone is essential to the success of VPT [12]. All laser types have some degrees of disinfecting ability which differs based on the penetration depth of various laser types [12]. This is particularly critical when the teeth are exposed due to caries. Since the bacterial penetration depth in dentin varies from 300 to 1000 µm [13], the depth of disinfection activity would be of paramount importance. Laser irradiation can also produce sufficient hemostasis and create an area of coagulative necrosis that besides achieving hemostasis, serves as a barrier to protect the pulp against direct contact with the pulp capping agent [14]. Below this thin necrotic layer, pulpal inflammatory cells and fibroblasts migrate and begin to form a dentinal bridge [12]. It is believed that laser irradiation minimizes hematoma and enables a close contact between the pulp tissue and pulp capping agent [15].

In some studies, it has been reported that the denatured layer formed by laser irradiation can delay the healing process of the pulp [16–18]. It appears that the thickness of the formed denatured layer is highly important in this regard. As the laser irradiation intensity increases, the thickness of the denatured layer increases which as well postpones pulp tissue recovery and leads to delayed healing [16, 17].

There are controversial clinical and radiographic reports regarding the efficacy of lasers in different treatment procedures related to vital pulp therapy. The majority of review studies focused on specific limited aspects of such treatments: therefore, this article aimed to review more broadly, the clinical and radiographic results of different types of vital pulp therapy with laser application in both primary and permanent teeth.

### Scope of the review

In our recently published review, we extensively explored the histological effects of lasers in vital pulp therapy [19] and this article provides a comprehensive review of the clinical and radiographic results of laser application in different types of vital pulp therapy. After description of the application of different laser types in various stages of vital pulp therapy, clinical and radiographic criteria for success and failure in primary and permanent teeth are elaborated separately and based on these criteria, the results of studies conducted with each laser type are presented.

### Search strategy

Two independent researchers conducted an electronic search of Scopus, MEDLINE, Web of Science and Google Scholar databases during 2000–2023. Different combinations of words including Laser, Erbium: Yttrium -Aluminum- Garnet (Er:YAG*),* Erbium,Chromium: Yttrium-Scandium-Gallium-Garnet (Er,Cr:YSGG), Neodymium-Doped: Yttrium Aluminum Garnet (Nd:YAG), Diode, Low-level laser therapy, CO_2_, Pulp, vital pulp treatment, vital pulp therapy, treatment outcome, radiography and similar phrases defined in relevant papers were used. In addition, cited references of included articles were assessed in search of other possible related articles. Articles written in languages other than English, were only included if the abstract was in English and encompassed the key information.

### Inclusion and exclusion criteria

**Fig. 1:**
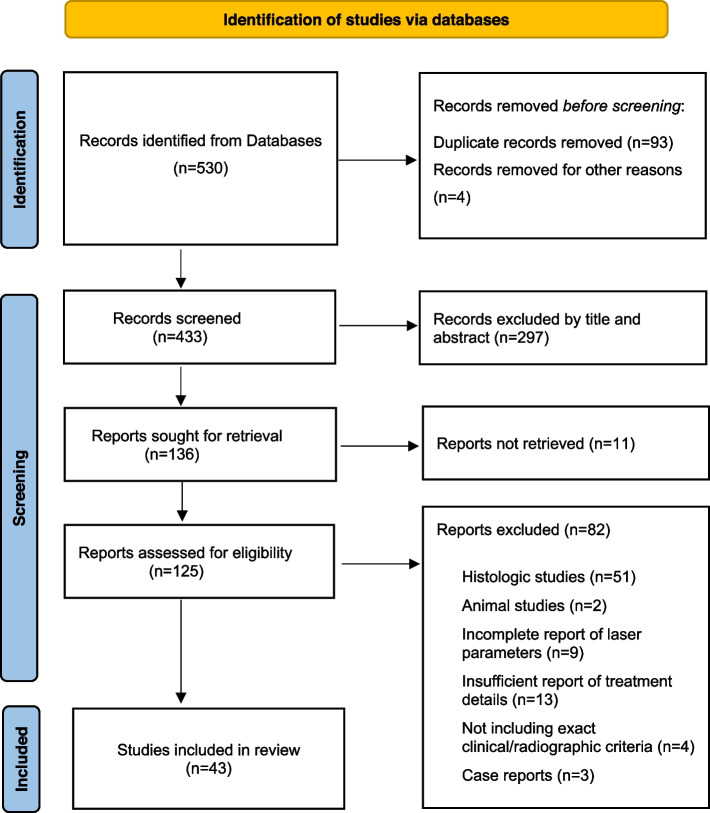
Search strategy and PRISMA flow diagram

**Fig. 2:**
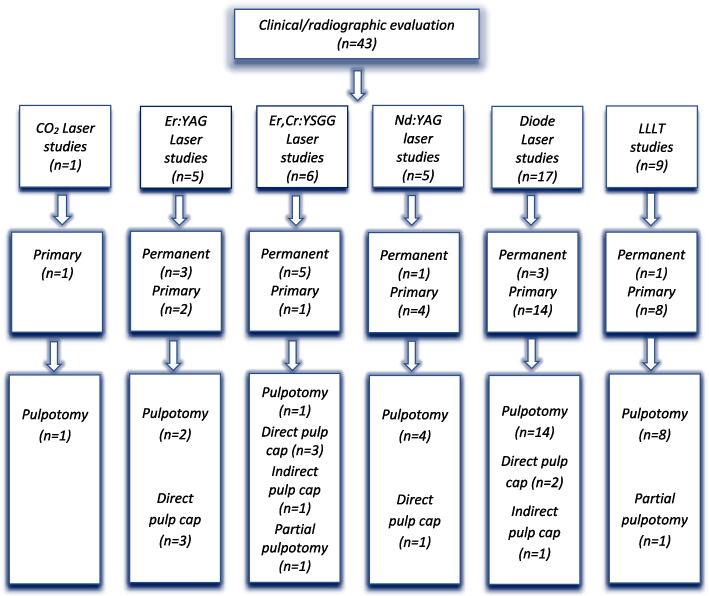
Classification of included studies

Only human studies on laser application in vital pulp therapy were included that directly mentioned laser setting parameters and success/failure criteria. Additionally, since this study concentrates on the clinical and radiologic assessment of VPT, all histologic and animal studies as well as review articles were excluded. Source selection and classification are presented in Figs. 1 and 2.

The success/failure criteria for VPT according to the literature are discussed below:

### Treatment success/failure in primary teeth

#### Clinical criteria

According to the literature, in assessment of clinical success of laser therapy in primary teeth, the success criteria include asymptomatic tooth (no sensitivity to percussion, palpation, or pressure, and no spontaneous pain), absence of sinus tract, absence of swelling and abscess, and absence of abnormal mobility [20–23]. Some other studies also included absence of premature loss of tooth as a clinical success criterion for primary teeth [23–28].

#### Radiographic criteria

Absence of radiolucency at the furcation area or periapical region, and absence of internal and external root resorption in primary teeth are among the treatment success criteria [23, 29, 30]. Some other studies also included no damage to permanent tooth bud as a radiographic treatment success criterion [27, 31]. Some studies considered calcification a criterion for success in radiography [32, 33] while others considered it a criterion for failure [34]. The failure criteria for vital pulp therapy have also been cited as periodontal ligament widening [20, 24, 26, 28–30, 34–39] and loss of lamina dura integrity [40]. Shaikh et al., in their study, scored the clinical and radiographic criteria separately by using a 4-point scale. In addition to the usual clinical parameters used in other studies, gingival inflammation and periodontal pockets were considered as distinguishing clinical criteria in this study [38].

### Treatment success/failure in permanent teeth

#### Clinical criteria

Absence of sensitivity to percussion and functional forces, absence of spontaneous pain, absence of sinus tract, swelling, and abscess, and absence of abnormal tooth mobility are among the clinical success criteria for permanent teeth [12, 41–43]. Some other studies also used cold and heat pulp tests and electric pulp test to assess the tooth responsiveness [12, 17, 41–46].

#### Radiographic criteria

Absence of radiolucency and any osseous change in the periapical or furcation areas, and absence of resorption are generally considered indicators of radiographic success [45]. The periodontal ligament widening [42, 45, 47] and loss of lamina dura [47] were also used as radiographic failure criteria. Partially or completely closure of apex is considered a success criterion when the experiments were conducted on premature permanent teeth [47, 48]. In a study by Sharma et al., [41] the radiographic success of indirect pulp cap was evaluated by measuring the distance between the uppermost point of the pulp horn and the cavity base.

## Results

### Low-level laser therapy (LLLT)

LLLT was mostly used for pulpotomy of primary teeth. There were no clinical or radiographic benefits associated with LLLT compared with formocresol (FC) in vital pulp therapy of primary teeth in the majority of the included studies [35, 37, 49]. In a study by Fernandes et al., pulpotomy with FC showed superior radiographic success to LLLT after 6 to 18 months [32] while in another study by Yavagal et al., pulpotomy with LLLT showed a significantly higher radiographic success rate in comparison with FC after 9 months [50].

In pulpotomy treatments, the use of LLLT just before the application of calcium enriched mixtures (CEM) and calcium hydroxide (CH) did not enhance the radiographic or clinical success rate [20, 36]. It is worth to highlight that, applying an appropriate material as the pulp capping agent in VPTs is essential. Therefore, laser therapy alone and without the application of pulp capping agent may jeopardize dentinal bridge formation [32, 42]. For instance, Fernandes et al. [32] demonstrate that the sole application of LLLT, without a pulp capping agent, did not lead to dentinal bridge formation. Table 1 shows the included studies by LLLT.

**Table 1:** Findings of clinical and radiographic studies on low-level laser therapy (LLLT) in vital pulp therapy

Author	Study type	Number of teeth	Treatment type	Materials used in vital pulp therapy	Laser characteristics	Follow-up duration	Results
Fernandes et al. [32]	Human in vivo	60 mandibular primary molar teeth	Pulpotomy	FC + ZOE + RMGI CH + ZOE + RMGI LLLT + CH + ZOE + RMGI LLLT + ZOE + RMGI	LLLT In-Ga-AlP 660 nm 2.5 J/cm^2^ 10 mW 50–60 Hz 10 s Contact mode	6,12,18 months	Clinical success rate: 100% in all groups over the follow up period Radiographic success rate after 6,12 and 18 months:(sig.) FC: 100%,100%,100% CH: 60%,50%, 66.7% LLLT: 80%,80%,73.3% LLLT + CH:85.7%,78.6%,75% Number of teeth with internal resorption: FC < LLLT + CH < LLLT < CH Hard tissue barrier was seen in CH groups: CH < LLLT + CH Pulp calcification was seen in all groups: LLLT + CH < FC < CH < LLLT No hard tissue barrier was observed in the FC and LLLT groups
Uloopi et al. [39]	Human in vivo	40 primary molar teeth	Pulpotomy	MTA + GI + SS crown LLLT + GI + SS crown	LLLT Diode laser 810 nm CW 2 J/cm^2^ 10 s	3,6,12 months	Overall (Clinical&Radiographic) success rate after 3,6,12 months:(non sig.) MTA:94.7%,94.7%,94.7% LLLT:95%,85%,80%
Alamoudi et al. [35]	Human in vivo	106 primary molar teeth	Pulpotomy	FC + IRM + SS crown LLLT + IRM + SS crown	LLLT Diode laser 810 nm 6.7 J/cm^2^ 40 s Non-contact (2 mm) 3 W Continuous mode	6,12 months	Clinical success rate after6,12monthes:(non sig.) FC:98%, 96.1% LLLT:98%, 96.1% Radiographic success rate after 6,12 months: FC:98%, 98% LLLT:100%, 100%
Golpayegani et al. [37]	Human in vivo	46 primary molars	pulpotomy	LLLT + ZOE + reinforced ZOE + SS crown FC + ZOE + reinforced ZOE + SS crown	LLLT Diode laser 632 nm Continuous mode/2.5 s 4 J/Cm^2^ Non contact (2 mm)	6,12 months	Clinical success rate after 6,12 months: LLLT:100%,89% FC:100%,100% Radiographic success rate after 6,12 months: LLLT:89%, FC:100%,100%
Ansari et al. [36]	Human in vivo	160 primary molar teeth	pulpotomy	FC + Zonalin + SS crown FS + Zonalin + SS crown CEM + Zonalin + SS crown LLLT + CEM + Zonalin + SS Crown	LLLT Diode laser 632 nm Continuous mode 4 J/cm^2^ 135 s	6,12 months	Clinical success rates after 6,12 months: (non sig.) FC:100%-100% FS:97.5%-95% CEM:100%-97.5% LLLT + CEM:100%-100% Radiographic success rate after 6,12 months: (non sig) FC:100%-100% FS:97.5%-92.5% CEM:100%-95% LLLT + CEM:100%-100%
Yavagal et al. [50]	Human in vivo	68 primary molars	pulpotomy	FC + ZO (Eugenol-free) + GI + SS crown LPBM(LLLT) + GI + SS crown	LLLT Ga-Al-As Diode laser 660-nm 36 mW Non-contact mode 4 min 8.64 J/cm^2^	9 months	Clinical success rate: (non sig.) FC:97.05% LLLT:94.1% Radiographic success rate: (sig.) FC:58.82% LLLT:94.1%
Nadhreen et al. [49]	Human in vivo	106 primary molars	pulpotomy	LLLT + IRM + SS crown FC + IRM + SS Crown	LLLT Diode lassr 810-nm 4 J 6.7 J/cm^2^ Non-contact mode 40 s 1-50KHZ	3,9 months	Clinical success rate after 3, 9 months: (non sig.) LLLT:98%,98% FC:98%,98% Radiographic success rate after 9 months: (non sig.) LLLT:100% FC:98%
Ebrahimi et al. [20]	Human in vivo	63 primary molar teeth	Partial pulpotomy	MTA LLLT (Low power diode laser) + MTA Diode laser(high power) + MTA	LLLT Diode laser (low power) 660-nm 200mW Diode laser (high power) 810-nm 1W	6,9,18 months	Clinical success rate: (non sig.) MTA:100%,100%,100% LLLT + MTA:100%,100%,100% Diode laser + MTA:95.2%,95.2%,87.5% Radiographic success rate after 6,9,18 months:(non- sig) MTA:90.5%,90.5%,87.5% LLLT + MTA:100%,95.2%,88.2% Diode laser + MTA:85.7%,76.2%,68.7%
Kaya et al. [51]	Human in vivo	172 primary molars	Pulpotomy	LLLt(PBMT)+CH MTA CH FC	LLLT Diode laser 820-nm 10mW 2.5 J/cm^2^ 12s	6,12 months	Clinical & radiographic success rate after 12 months: LLLT(PBMT) + CH:87%,73% MTA:97%,95% CH:71%,45% FC:97%, 92%

### Diode laser

This near infrared laser has higher efficacy for dentin disinfection and hemostasis compared with erbium lasers or CO_2_ laser types_._ which is related to its higher penetration depth [12]. In this regard, the diode laser is irradiated in non-focusing continuous wave mode with 0.4–0.5 W power for a maximum of 5–10 s [52].

This laser was used during pulpotomy treatment of primary teeth in most studies [29, 31, 33, 34]. Other studies have used this laser during direct [12, 31, 43] and indirect pulp capping treatment [41] as well as pulpotomy for apexogenesis purposes [52]. Most studies indicate that [29, 31, 33, 36, 38], diode laser did not show any significant benefit over FC (which is considered as the gold-standard for VPT of primary teeth) [53] or ferric sulfate (FS) [29, 34]. However, in a study by Gupta et al., [54] the clinical and radiographic success of diode laser for pulpotomy of primary teeth was reported to be 100% at 12 months, and significantly higher than that of FS and electrosurgery. In some studies, diode laser was applied on the pulp prior to pulp capping with MTA [27, 28, 30, 55], Biodentine (BD) [41], resin-modified glass ionomer [12, 41] or resin-modified calcium silicate paste (TheraCal) [43]. Studies with laser irradiation prior to the application of MTA in pulpotomy of primary teeth, did not show superior clinical and radiographic results [27, 28, 55]. Studies with laser irradiation prior to pulp capping with TheraCal increased the clinical success of treatment [43]. Sharma et al. showed that application of diode laser prior to indirect pulp capping with resin-modified glass ionomer or Biodentine did not increase the dentinal bridge thickness significantly [41].

In a slightly different study [25], three methods of laser irradiation, placement of a sterile cotton pellet, and sodium hypochlorite wash were used for hemostasis in pulpotomy of primary teeth. It was concluded that laser had a superior clinical and radiographic efficacy compared with sodium hypochlorite for hemostasis in pulpotomy treatment at 24 months. They also indicated that pulpotomy by attending dentists had a significantly higher success rate than by postgraduate students.

In a study comparing diode laser pulpotomy with simvastatine (a novel medicament in regenerative treatments) [40] no significant superiority in laser application was found.

Table 2 shows the included studies by diode laser pulp therapy.

**Table 2:** Findings of clinical and radiographic studies on diode laser in vital pulp therapy

Author	Study type	Number of teeth	Treatment type	Materials used in vital pulp therapy	Laser characteristics	Follow-up duration	Results
Yadav et al [34]	Human in vivo	45 primary molar teeth	Pulpotomy	FS + ZOE + GI Electrosurgery + ZOE + GI Laser + ZOE + GI	Diode 810-nm 3 W CW 2–3 s Contact Mode	1,3,6,9 months	The overall clinical success rate: (non-sig) FS:86.6% ES:100% Laser:100% The overall radiographic success rate: (non sig) FS:80% ES:80% Laser:80% Most common radiographic findings of failure: Internal resorption
Yazdanfar et al [12]	Human in vivo	10 permanent teeth 10 permanent Ant&Post. teeth	Pulp capping	RMGI + Composite Laser + RMGI + composite	Diode Laser (Hemostasis) 808- nm CW 1.5 W Contact Mode + (Decontamination) 808-nm CW 1 W Contact Mode	1,6,12 months	Clinical success rate after 1 year: (sig) RMGI:60% Laser:100% The failed treatments were related to youngest patients which reflects the importance of microbial free environment than age
Durmus et al. [29]	Human in vivo	120 primary molar teeth	pulpotomy	FC + ZOE + GI + SS crown FS + ZOE + GI + SS crown Diode laser + ZOE + GI + SS crown	Diode laser 810-nm 1.5 W 30 Hz 50 mJ 10 s Non contact (1-2 mm) mode/air cooling (without water)	1,3,6,9,12 months	Clinical success rate after1,3,6,9,12 months as follows: (not sig.) FC:100%-100%-100%-97.5%-97.5% FS:100%-100%-97.5%-95%-92.5% Laser:100%-100%-100%-100%-100% Radiographic success rate for 1,3,6,9,12:(non sig.) FC:100%-97.5%-92.5%-90%-87.5% FS:100%-95%-87.5%-84.6%-79.5% Laser:100%-95%-87.5%-87.5%-75%
Gupta et al. [54]	Human in vivo	30 primary molars	pulpotomy	FS Electrosurgery Laser	Diode laser 980 nm 3W Continuous pulse mode Contact mode 4 J/cm^2^ 31 s	3,6,9,12 months	Clinical & radiographic success rate after3,6,9,12 months: (sig.) FS:80%-80%-80%-80% ES:80%-80%-80%-80% Laser:100%-100%-100%-100%
Joshi et al. [31]	Human in vivo	40 primary teeth	pulpotomy	Diode laser + ZOE + GI FC + ZOE + GI	Diode laser 980-nm Continuous mode 2 s Contact mode 1.5 W	3,6,12 months	Clinical success rate after3,6,12 months: (non sig.) Diode laser:100%,100%,100% FC:100%,100%,100%,100% Radiographic success rate after3,6,12 months: (non sig.) Diode laser:100%,94.4%,78.8% FC:94.4%,78.8%,57.8%
Niranjani et al. [26]	Human in vivo	60 primary molars	pulpotomy	MTA + ZOE + SS crown Laser + ZOE + SS crown BD + ZOE + SS crown	Diode laser 810 nm Contact mode 1.5W 2 s	3,6 Months	Clinical and radiographic success rate after 6 monthes in MTA group was 100%. (non sig.)
Pratima et al. [30]	Human in vivo	40 primary molars	pulpotomy	Laser + MTA + ZOE + SS crown Laser + ZOE + SS crown	Diode laser 980-nm 2.5–3 W Contact mode	3,6,12 months(clinically) 6,12 months(radiographically)	Clinical success rate after 3,6,12 months: (non sig.) Laser + MTA:100%,100%,100% Laser + ZOE:94%,94%,94% Radiographic success rate after 6,12monthes:(non sig.) Laser + MTA:100%,100% Laser + ZOE:94%,94%
Saltzman et al. [27]	Human in vivo	52 primary teeth	pulpotomy	Laser + MTA + GIC + SS crown FC + ZOE + SS crown	Diode laser 980-nm 3 W Continuous mode Contact mode	2.3,5.2,9.5,15.7 months	Radiographic success rate after 2.3,5.2,9.5,15.7 months: (non sig) Laser + MTA:95.8%,94.7%,77.8%,71.4% (overal:70.84%) FC + ZOE:100%,100%,94.7%,84.6%,(overall:87.5) All teeth in both treatment groups were regarded as clinically successful at each follow up period Clinical-pathological evaluation showed that apart from expected histologic changes, iatrogenic errors were present in all teeth that were considered as failure
Yazdanfar et al. [43]	Human in vivo	20 permanent teeth	Pulp Cap	TheraCal (Resin Modified Calcium Silicate Paste) + Scotchbond + Composite Laser + TheraCal + Scotchbond + Composite	Diode Laser 808-nm 1.5W CW Contact Mode 190.98 W/cm^2^ 381.97 J/cm^2^	1,3,6 months	Clinical assessment: All The teeth in both groups remained vital after 6 monthes All the teeth in TheraCal group showed slight sensitivity to cold test. (Significantly more than Laser group) Radiographic assessment: There was not significant difference among groups The Laser group showed higher thickness and better integrity in regard to reparative dentin formation
Pei et al. [33]	Human in vivo	90 primary molar teeth	pulpotomy	FC + IRM + SS crown Laser + IRM + SS crown	Diode laser 915-nm 2 W 100 Hz Contact mode 1 s (3times)	3,6,12 months	Clinical success rate after 3,6,12 months: (non sig.) Laser:100%,96.8%,92.9% FC:100%,97.1%,90.9% Radiographic success rate after 3,6,12 months:(non sig.) Laser:100%,90.3%,78.6% FC:100%,91.4%,72.7%
Swarnalatha et al. [55]	Human in vivo	40 primary teeth	pulpotomy	MTA + GIC + SS crown Laser + MTA + GIC + SS crown	Diode laser 810-nm Continuous mode Contact mode 1.5 W 2 s	3,6,9 months	Clinical success rate after3,6,9monthes: MTA:90%,84.21%,88.23% Laser:95%,94.74%,99.44% Radiographic success rate after 3,6,9monthes: MTA:85%,84.21%,82.3% Laser:90%,89.47%,88.89%
Kuo et al [25]	Human in vivo	145 primary molars	pulpotomy	Laser (hemostasis) + ZOE + GI + SS crown/Composite resin NaOCl (hemostasis) + ZOE + GI + SS crown/composite resin Sterelized dry cotton pellet (hemostasis) + ZOE GI + SS crown/Composite resin	Diode Laser 970-nm 3 W 5 Hz Water cooling	6,12,18,24 months	Clinical success rate after 6,12,18,24 months: Laser:100%,100%,100%,100% NaOCl:98.8%,96.2%,94.4%,88.9% No medication:100%,100%,100%,100% (Sig. difference between Laser&NaOCl after 24 months) Radiographic success rate after after 6,12,18,24 months: Laser:100%,97.6%,97.4%,90.9% NaOCl:98.8%,85.7%,82.2%,65.7% No medication:100%,92.6%,92.6%,87.5% (Sig. difference between Laser&NaOCl after 12,18,24 months)
Sharma et al. [41]	Human in vivo	40 permanent molar teeth	Indirect pulp cap	RMGI + IRM Laser + RMGI + IRM BD + IRM Laser + BD + IRM	Diode Laser 980-nm 1 W CW Contact mode 10 s	3,6,12 months	Clinical assessment: All the teeth were vital and had positive response in regard to cold test and Electrical Pulp Test Radiographic assessment: The thickness of dentin deposited after 12 months: (non sig) RMGI:0.07 mm Lser + RMGI:0.10 mm BD:0.25 mm Laser + BD:0.32 mm
Aripirala et al. [40]	Human in vivo	100 Primary molar teeth	pulpotomy	Diode laser + RMGI + SS Crown SV(Simvastatine gel) + RMGI + SS Crown	Diode laser 940-nm 2 W 4 J/cm^2^ 70–80 Hz Gated pulse mode (for pulp amputation) CW (for hemostasis)	3,12 months	Clinical & radiographic success rate after 12 months:(non- sig) Laser:76.1%,52.1% SV:80.4%,65.2%
Satyarth [28]	Human in vivo	40 primary teeth	pulpotomy	MTA Laser + MTA	Diode Laser 810 nm CW 1.5W Contact mode	3,6,9 months	Clinical success rate after 3,6,9 months: MTA:90%,84.21%,88.23% Laser:95%,94.74%,94.44% Radiographic success rate after 3,6,9 months: MTA:85%,84.21%,82.3% Laser:90%,89.47%,88.89%
Shaikh et al. [38]	Human in vivo	40 primary teeth	pulpotomy	FC Laser	Diode Laser 810-nm 1.5 W Continuous Mode Contact Mode 10 s	1,3,6,9 months	There was no significant clinical and radiographic difference between the two groups
Ansari et al.[24]	Human in vivo	40 primary teeth	pulpotomy	Laser + Reinforced ZOE + SS Crown FC + Reinforced ZOE + SS Crown	Diode Laser 810-nm Non-contact 10 W 20 Hz	6,12 months	Clinical success rate after 6,12 months: (non sig) FC:100%,100% Laser:100%,100% Radiographic success rate after 6,12 months: (non sig) FC:100%,100% Laser:95%,90%

### Nd:YAG laser

Since Nd:YAG laser as a near infrared laser, has a higher penetration depth than erbium and CO_2_ lasers [12], it would have more effective disinfecting ability and hemostatic action [43, 56]. Therefore, it should be preferably used with lower energies of approximately 50 mJ to 75 mJ with 10 Hz frequency, and 100 µs pulse in non-focusing mode to safely benefit from its antimicrobial effects [52].

According to the limited research that has been performed on this type of laser, pulpotomy with Nd:YAG laser induced greater pulpal calcification than FC [57]. Long-term (66 months) follow-ups revealed that this laser was significantly more effective than FC [22]; however, shorter (12 months), follow-ups did not yield similar results [23]. Nd:YAG laser was differently used in some studies. Furze et al. [48] evaluated success rate of pulpotomy in primary and young permanent teeth. They used Er:YAG laser for dentin preparation and caries removal, and then used Nd:YAG laser for coagulation and sterilization. They used different pulp capping agents and concluded that irrespective of the type of capping agent, the success of this treatment was 100% in permanent teeth, which was insignificantly higher than that in primary teeth (95.3%). In the study by Gunaydin et al., [44] all permanent teeth underwent pulp capping with MTA and then one group of them was subjected to Nd:YAG laser irradiation. The laser group reported lower level of postoperative pain and discomfort after 7 days. However, radiographic and clinical outcomes did not differ significantly between the two groups.

### Er:YAG or Er,Cr:YSGG lasers

As mentioned before, of different laser types, currently, only erbium lasers have the potential for use in cavity preparation because erbium lasers are absorbed by water and hydroxyapatite due to their specific wavelength. Erbium is the only laser type that can be used for hard tissue preparation with minimal pulpal thermal damage [8, 42]. Depending on the case, Er:YAG or Er,Cr:YSGG laser may be used along with the handpieces for caries removal [3]. For cavity preparation and caries removal with these laser types, lasers should be used with energy levels not more than 150 mJ and with 15 to 20 Hz frequency in short (100 to 300 µs) pulse durations under air/water spray [52].

According to studies, application of Er,Cr:YSGG [44] or Er:YAG [42] laser prior to pulp capping with CH [42, 45] or TheraCal [45] significantly increased the overall clinical and radiographic success of direct pulp capping; however, its application prior to pulp capping with MTA made no significant change in success of partial pulpotomy or direct pulp cap treatment [46, 47]. Furthermore, it was demonstrated that the clinical and radiographic results of pulpotomy of primary teeth with Er:YAG or Er,Cr:YSGG laser had no significant difference with the results of pulpotomy with FS, CH, FC [21, 58], biodentine and MTA [59].

## *CO*_*2*_* laser*

CO_2_ laser has wavelengths of 9300 and 10,600 nm and has optimal absorption in water and hydroxyapatite [59]. This laser has been used with different exposure settings in the literature [16–18].

It has been observed that the majority of CO_2_ laser energy is absorbed at 0.1 to 0.2 mm depth, and the thickness of denatured layer after application of this laser type is less than 0.5 mm. Thus, application of this laser is suitable for hemostasis in the exposed pulp [16]; However, it should be noted that the effect of CO_2_ laser, similar to other laser types, highly depends on its intensity such that a higher laser output results in a thicker denatured and coagulation layer, and as mentioned, delays optimal tissue healing [16]. Studies on the application of CO_2_ laser for VPT are sparse. The clinical and radiographic success rate of laser for pulpotomy of primary teeth is reportedly 98% and 91%, respectively [59].

## Discussion

Studies on the effects of laser VPT have reported controversial results. The possible causes for this controversy are briefly discussed. In order to compare the results of VPT, the following parameters should be taken into account:

### Laser settings

Laser power, frequency, and irradiation time are among the important parameters that have been variable in different studies. Fluence of laser is another important parameter in LLLT, such that very high or very low doses cannot exert optimal biological effects [59]. It is a high-standard parameter to reveal the amount of energy received by the cells in Joule/square centimeter (J/cm^2^) [59].

### Duration of follow-up

The required duration of follow-up for patients who have undergone VPT has not yet been determined; however, it appears that since after 21 months, the success rates were similar,

this time period would be a suitable duration for the follow-up of patients and determination of prognosis of pulp capping treatments [60].

According to a meta-analysis, duration of follow-up is an important parameter in reporting the results of VPT [61]. For instance, Odabas et al., [23] and Liu et al., [22] both used similar laser types with similar settings; Odabas et al. [23] followed up the patients for one year while Liu et al. [22] followed up the patients for 5 years. Liu et al. found a significant difference between the laser and FC groups; however, this difference was not significant in the study by Odabas et al. One possible reason for variations in the results of apparently similar studies can be different durations of follow-up.

Unfortunately, to the best of our knowledge, no other study with a long-term follow-up is available in this regard, and the studies by Liu et al. [22, 57] were the only studies that assessed the long-term success of laser pulpotomy. Matsu et al. concluded that 3 months would be enough to determine the tentative prognosis of treatment since the success rate was the same at the 3- and 18-month follow-ups in their study [60]. However, they mentioned that teeth subjected to VPT should be followed up for 21 months because the treatment success increased after 21 and 24 months.

### Tooth restoration after pulpotomy

In a study by Saltzman et al. [27], it was concluded that iatrogenic errors, such as ill-fitting stainless- steel crowns, low MTA thickness, and coronal pulp residues not completely removed during pulpotomy, appeared to play a role in the failure of treatment; This was, found to be especially true in laser pulpotomy which has higher technique sensitivity than the conventional pulpotomy with FC [27]. Guelmann et al. [62] showed that resin-modified glass ionomer can provide optimal marginal seal and excellent retention and can serve as a suitable alternative to stainless steel crowns after pulpotomy of primary teeth. Although Croll and Killian [63] suggested stainless steel crowns as the treatment of choice for pulpotomized primary teeth. Holan et al. [64] proposed restoration of class I amalgam restorations given that teeth are expected to undergo physiological exfoliation within the next 2 years; their reasoning held that, no significant difference was noted in their success rates comparing the two types of restorations. Although most studies did not assess the success rate based on the type of final restoration, it should be noted that in some studies, such as the one by Odabas et al., [23] all failed teeth in both laser and control groups had been restored with stainless steel crowns.

Some studies recommended laser irradiation prior to the application of pulp capping agents such as MTA, and CH in VPT [42, 45]. Zinc oxide eugenol (ZOE) is extensively used as a base in pulpotomized teeth due to its desirable anodyne and antibacterial properties. Moreover, ZOE provides an appropriate seal, and minimizes the risk of microleakage and subsequent infection [30]. According to the literature, direct contact of ZOE with the pulp tissue in laser pulpotomy compromises the success of treatment due to the release of eugenol, which can initiate chronic inflammatory reactions in dental pulp [53]. However, when the pulp tissue is fixed with a material such as FC, or capped with a capping agent, it would not be affected by eugenol [27, 54].

### Misdiagnosis of sound pulp tissue

The failures reported in VPT in the literature are due to several factors, one of which is clinical misdiagnosis and incorrect patient selection. For instance, dental pulps with chronic inflammation are incorrectly diagnosed as sound and are eventually reported as a case of treatment failure. According to Huth et al., [58] in the application of laser for VPT, correct pulp status diagnosis is more critical than pulpotomy with FS and FC. In this regard, Durmus et al., [29] also believed that the pulp status in pulpotomy with FC was not as important as that in pulpotomy with FS and laser.

### Success and failure criteria

Different studies use different criteria for success/failure in VPT, and this can make a difference in the outcome. As an example, in the study by Durmus et al., [29] the main radiographic failure criterion considered was periodontal ligament widening, although patients did not show any pathological progress or clinical symptom at the end of the 12-month follow-up. In fact, a higher percentage of radiographic failure is reported in studies that do not differentiate between radiographic osseous and clinical changes, whereas many studies did not consider the periodontal ligament status as one of the failure criteria [22, 44, 57]. Alamoudi et al. [35] discussed that internal resorption in primary teeth should not be necessarily considered as a failure criterion given that it remains stable during repeated follow-ups, and does not cause root perforation, adjacent bone loss, clinical symptoms, or injury to the permanent successors [35, 65]. Calcific metamorphosis is another radiographic criterion which was considered as a success criterion in some [32, 33, 55] and failure criterion in some other studies [34]. A few others did not consider this criterion at all [27, 45, 58]. This parameter also plays an important role in the existing controversy in the results. It may be discussed that pulpal calcification should not be considered as a radiographic failure criterion since it indicates deposition of tertiary reparative dentin, and subsequent root canal stenosis or obliteration due to the activity of odontoblast-like cells, and highlights pulp vitality [32, 65].

It is also worth noting that some studies take both clinical and radiographic success into account as an overall score rather than assessing them separately. For instance, Uloopi et al., who calculated the overall rate of clinical and radiographic success and failure [39], reported lower success rate for laser therapy than Alamoudi et al., [35] who reported success and failures with separate criteria.

### Effect of age

No consensus has yet to be reached on the effect of age on the success/failure of VPT. Some studies believe that patient’s age should be considered in pulp capping treatment, and discuss that pulp tissue in young patients has higher reparative and regenerative potential compared with older individuals; whereas, many others did not find any evidence of lower success rate in older patients [45]. Olivi et al. [66] evaluated the effects of Er:YAG and Er,Cr:YSGG lasers on children and adults, and concluded that age and laser type did not affect the results of treatment. Furze et al. [48] concluded that application of Nd:YAG laser in pulpotomy of permanent teeth with immature apex did not yield a higher success rate than primary teeth. Studies on the effects of age on VPT are widely variable.

## Conclusion

Although current literature suggests laser may be proposed as an adjunct modality for some procedural steps in VPT, more research with standardized methodologies and criteria is needed to obtain more reliable and conclusive results. The goal is to develop evidence-based guidelines and protocols that will ultimately result in improved outcomes and prognoses for patients with VPT.

## Data Availability

The datasets used and/or analyzed during the current study are available from the corresponding author on reasonable request.

## Abbreviations

VPT: Vital pulp therapy
Er:YAG: Erbium-doped: yttrium -aluminum- garnet
Er,Cr:YSGG: Erbium,chromium: Yttrium-Scandium-Gallium Garnet
CO_2_: Carbon dioxide
Nd:YAG: Neodymium-doped: yttrium–aluminum-garnet
LLLT: Low-level laser therapy
FC: Formocresol
CEM: Calcium enriched mixtures
CH: Calcium hydroxides
FS: Ferric sulfate
BD: Biodentine
ZOE: Zinc oxide eugenol
